# Mapping the Somatic Mutation Landscape of Familial NF2-Related Schwannomatosis using Whole-Exome Sequencing

**DOI:** 10.7150/ijms.121550

**Published:** 2025-10-27

**Authors:** Fushu Luo, Liangqi Jiang, Yimin Pan, Haoyu Li, Jun Tan, Changwu Wu, Qing Liu

**Affiliations:** 1 Department of Neurosurgery, Xiangya Hospital, Central South University, Changsha, Hunan, China.; 2 National Clinical Research Center for Geriatric Disorders, Xiangya Hospital, Central South University, Changsha, Hunan, China.; 3 Clinical Research Center for Skull Base Surgery and Neuro-oncology in Hunan Province, Changsha, Hunan, China.

**Keywords:** Neurofibromatosis type 2, Whole-exome sequencing, Somatic mutation landscape, Genetic history

## Abstract

**Background:** Neurofibromatosis type 2 (NF2), currently more accurately named *NF2*-related schwannomatosis (*NF2-*SWN), is classified as a multiple tumor syndrome, caused by impaired expression of the merlin protein. Approximately 50% of affected individuals inherit a germline mutation from their parents, while reports on the somatic mutation landscape of other genes are infrequent.

**Aim:** To further explore the somatic mutations of *NF2-*SWN and provide a theoretical basis for the treatment of *NF2-*SWN.

**Design:** A retrospective study was conducted to follow up on *NF2-*SWN patients who underwent surgical treatment in the Department of Neurosurgery of Xiangya Hospital. Whole-exome sequencing (WES) was performed on patients with a clear family history.

**Methods:** This study compiled clinical data from 29 patients diagnosed with *NF2-*SWN, conducted WES on 7 patients with well-documented genetic histories, and subsequently analyzed their genetic mutations.

**Results:** Whole-exome sequencing identified frequent somatic mutations in genes such as TTN, FLG, CR2, and FSIP2. Missense mutations and C>T transitions were the most common alteration types.

**Conclusion:** TTN, CR2, FLG, and FSIP2 demonstrated elevated mutation frequencies in these familial *NF2-*SWN patients, indicating that these mutations may contribute to the development and progression of familial *NF2-*SWN.

## Introduction

Neurofibromatosis type 2 (NF2), now more accurately referred to as *NF2*-related schwannomatosis (*NF2-*SWN)^1^, is a rare autosomal dominant genetic disorder with an estimated incidence of 1 in 25,000 to 40,000 live births, affecting both genders equally^2^-^4^. It is characterized by the development of multiple neoplasms of the central and peripheral nervous systems, most notably bilateral vestibular schwannomas, which are present in nearly all affected individuals^5^. The phenotypic manifestation of *NF2-*SWN exhibits variability in terms of severity, age of onset, and tumor presentation, encompassing intracranial and/or spinal schwannomas, intracranial meningiomas, and ependymomas^2^, ^3^. Moreover, the specific type or locus of mutations within the *NF2* gene may affect the phenotypic diversity of tumor manifestations^6^.

The disorder arises from mutations in the *NF2* tumor suppressor gene located on chromosome 22q12, which encodes the protein merlin (MIM 607379), a critical regulator of cell proliferation and apoptosis through the Hippo tumor suppressor pathway^3^, ^4^, ^7^. Merlin plays a critical role in the formation of cell junctions, activation of anti-mitogenic signaling pathways, and inhibition of oncogenic gene expression, all of which are encoded by the *NF2* gene^7^, ^8^. More than 50% of individuals diagnosed with *NF2-*SWN have no family history of the disease, suggesting that they likely have de novo mutations in the *NF2* gene, which highlights the genetic heterogeneity of this disorder^9^, ^10^. Nonetheless, certain cases exhibit a distinct familial predisposition, and it remains uncertain whether the pattern of *NF2* mutations in these patients aligns with that of non-inherited *NF2-*SWN. Knudson's two-hit hypothesis offers an explanation for the onset of *NF2-*SWN^4^. Beyond mutations in the *NF2* gene, alterations in other genes may also contribute to the initiation of *NF2-*SWN^11^, ^12^. The disease is relentlessly progressive, with patients often developing multiple tumors over their lifetime, necessitating a multidisciplinary approach to management^4^.

Diagnosis of *NF2-*SWN is primarily based on clinical criteria, including the presence of bilateral vestibular schwannomas or a combination of other characteristic tumors and a family history of the disorder^4^. Advances in genetic testing have allowed for the identification of *NF2* mutations in both germline and somatic tissues, providing a more definitive diagnosis and enabling early intervention^13^, ^14^. Genetic studies have also revealed that the clinical phenotype of *NF2-*SWN can vary significantly, even within families, due to factors such as mosaicism and the timing of biallelic inactivation of the *NF2* gene^13^. This variability underscores the importance of personalized medicine in the management of *NF2-*SWN, as treatment strategies must be tailored to the individual tumor burden and clinical symptoms of each patient^4^, ^5^.

In this study, we conducted a retrospective analysis of the clinical and molecular pathological features of 29 patients diagnosed with *NF2-*SWN treated at the Department of Neurosurgery, Xiangya Hospital, China. Moreover, we reported novel related gene mutations in the exon sequencing results of patients with 7 cases of familial *NF2*-related schwannomatosis.

## Methods

### Patients

A total of twenty-nine *NF2*-related schwannomatosis patients diagnosed and treated surgically in the Department of Neurosurgery at Xiangya Hospital, Central South University between June 2013 and June 2023 were included in this study. All diagnoses were confirmed based on the updated diagnostic criteria for *NF2-*SWN (Table 1) Clinical data, imaging findings, pathological results, and other treatment-related information were retrieved from the hospital information system. The cohort consisted of 15 male and 14 female patients. A comprehensive family history review identified 7 individuals with a potential familial predisposition to *NF2-*SWN. Blood samples were collected from these patients for whole-exome sequencing (WES). This research was conducted in accordance with the principles outlined in the Declaration of Helsinki. The study was approved by the Medical Ethics Committee of Xiangya Hospital, Central South University (Approval number: 202407141), and written informed consent was obtained from all participants.

### DNA extraction and library construction

Genomic DNA was extracted from intracranial tumor tissue using Agilent SureSelect Human All Exon V6 kit. DNA quantification and integrity were determined by the Nanodrop spectrophotometer (Thermo Fisher Scientific, Inc., Wilmington, DE) and the 1% agarose electrophoresis, respectively. Human genomic DNA samples were captured using Agilent SureSelect Human All Exon v6 library (Agilent Technologies, USA) following the manufacturer's protocol. Briefly, the genomic DNA was sheared into short fragments using the kit's enzyme. The sheared deoxyribonucleic acid (DNA) was purified and treated with reagents supplied with the kit according to the protocol. Adapters from Agilent were ligated onto the polished ends and the libraries were amplified by polymerase chain reaction (PCR). The amplified libraries were hybridized with the custom probes. The DNA fragments bound with the probes were washed and eluted with the buffer provided in the kit. Then these libraries were sequenced on the Illumina sequencing platform (NovaSeq 6000, Illumina, Inc., San Diego, CA) and 150 bp paired-end reads were generated. The WES and analysis were conducted by OE Biotech Co., Ltd. (Shanghai, China).

### Whole-exome sequencing analysis

The raw data was compiled in fastq format. To obtain high-quality sequences suitable for subsequent analysis, the raw reads were preprocessed using fastq (Version: 0.20.0). Initially, adapter sequences were removed, and bases with an average base quality score below 20 were filtered out. Additionally, reads containing ambiguous bases or shorter than 75 bp were also filtered. The clean reads were then aligned to the human reference genome (GRCh37) using BWA (Version: 0.7.17). SAMtools (Version: 1.9) was utilized to convert the mapped reads to the appropriate format. Using paired blood sample sequencing or the 1000 Genomes Project data as a reference^15^, somatic single nucleotide polymorphisms (SNPs) and insertions/deletions (INDELs) were identified. When the data from the 1000 Genomes Project was used as a reference, variants with a population frequency greater than 0.1% in the database were filtered out to minimize the inclusion of common germline polymorphisms. Similar to previous studies^16^, ^17^, the R package “maftools” was used to generate a summary plot of single nucleotide variation (SNV), while the R package “ComplexHeatmap” was utilized to visualize the overall somatic alterations in an OncoPrint plot^18^.

## Results

### The clinical characteristics of *NF2*-SWN

**Figure 1:**
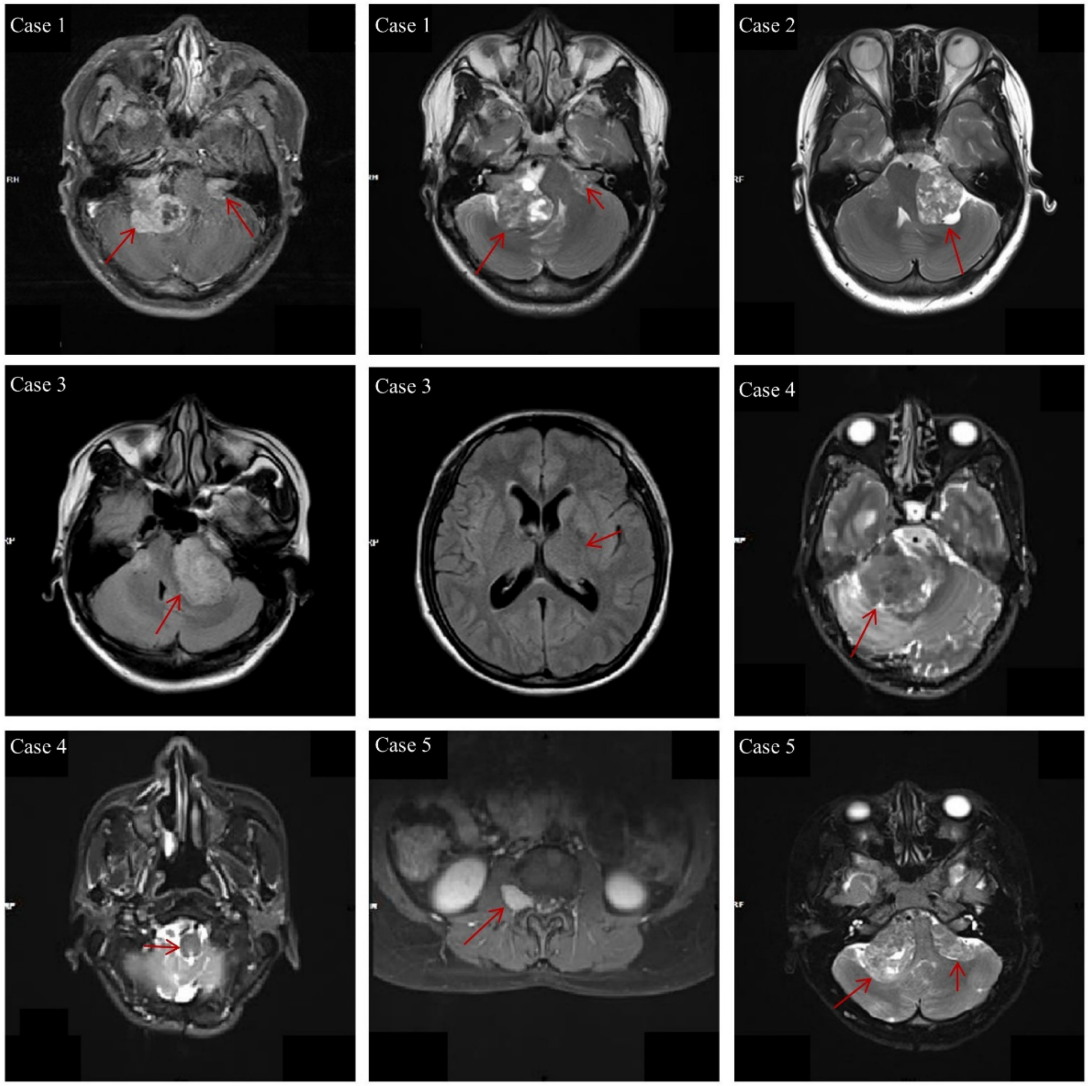
** The typical radiological findings of intracranial tumors in NF2-SWN patients.** Case 1: Bilateral vestibular schwannomas; Case 2: Unilateral vestibular tumor, first-level relatives suffer from NF2-SWN; Case 3: Multiple meningiomas and unilateral vestibular schwannoma; Case 4: Heterozygous schwannomas (schwannoma and perineurium); Case 5: Vestibular schwannoma with intraspinal and subcutaneous masses.

The clinical characteristics of the 29 *NF2-*SWN patients are listed in Table 2. The age of onset for almost all *NF2-*SWN patients was less than 40 years (93.1%), and most patients presented with tinnitus and hearing loss when visiting the hospital (72.4%). The majority of tumors were located in the cerebellar horn region (subtentorial, 96.7%), which is a primary cause of hearing loss among these patients. Furthermore, most patients exhibited multiple intracranial tumors (93.1%), with pathological results indicating that schwannomas were the most common tumor type (79.3%). Notably, patients with multiple tumors predominantly presented with a combination of schwannomas and meningiomas, which supports the second diagnostic criterion for *NF2-*SWN. Figure 1 demonstrates the typical radiological findings of intracranial tumors in *NF2-*SWN patients, including those with schwannomas combined with meningiomas.

**Figure 2:**
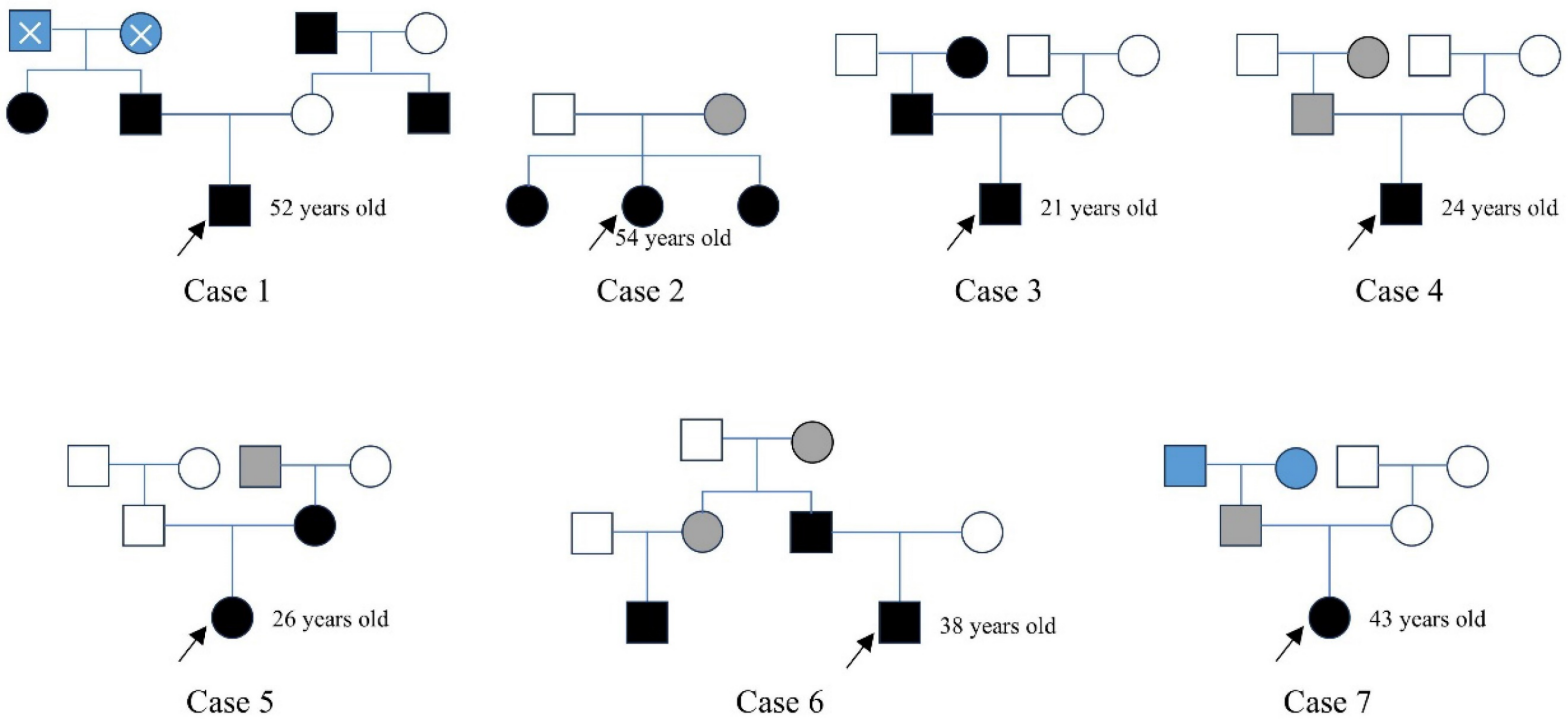
** The family maps of patients with inherited *NF2*-SWN.** In the pedigrees, black symbols represent individuals diagnosed with *NF2-*SWN, white symbols indicate unaffected individuals, grey symbols denote suspected cases, and blue symbols indicate individuals with unknown disease status. A cross-symbol marks deceased individuals. Squares represent males, and circles represent females. A black arrow highlights the proband, i.e., the individual who sought medical care at our hospital.

For *NF2-*SWN patients with a suspected family history, we conducted follow-up investigations into their family backgrounds and constructed family pedigrees (Figure 2). In Case 1, the patient's father, aunt, uncle, and maternal grandfather were all diagnosed with *NF2-*SWN; however, his paternal grandparents had passed away, and their *NF2-*SWN status could not be confirmed. In Case 2, a 54-year-old female patient and her two sisters were diagnosed with *NF2-*SWN, while their mother exhibited hearing loss symptoms but lacked definitive imaging findings, thus being classified as a suspected *NF2-*SWN case. In Case 3, both the patient's father and grandmother were confirmed *NF2-*SWN patients, clearly indicating a familial inheritance pattern. In Case 4, a 24-year-old male patient reported that his father and grandmother exhibited hearing loss symptoms, but no further clinical evidence supported an *NF2-*SWN diagnosis, so they were also categorized as suspected cases. In Case 5, both the patient and her mother were diagnosed with *NF2-*SWN, and it was reported that her maternal grandfather had suffered from acoustic neuroma (no medical records available; marked as a suspected case). In Case 6, the patient's father and cousin were confirmed to have *NF2-*SWN, and he reported that his aunt and grandmother had experienced hearing loss, although no relevant diagnostic reports were available. In Case 7, the female patient reported unilateral hearing loss in her father, while the health status of her grandparents remained unknown. Analysis of the constructed pedigrees did not identify any specific genetic transmission patterns, such as maternal or paternal inheritance.

### The whole-exome sequencing analysis of familial *NF2*-SWN

**Figure 3:**
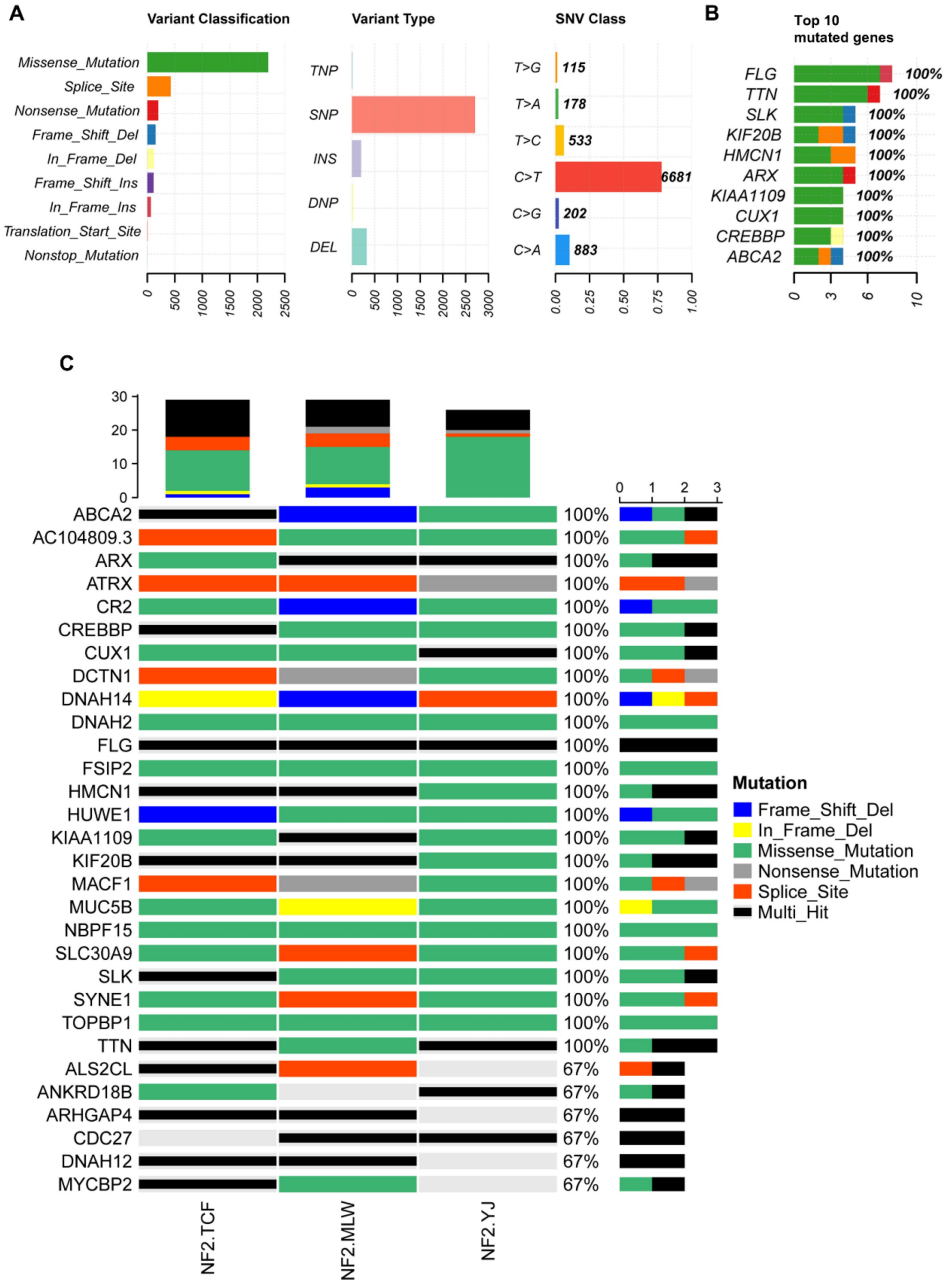
** The somatic mutation landscape of three *NF2*-SWN patients with paired blood samples. A**, Landscape and counts of mutation types. **B**, The top 10 genes with the highest mutation counts. **C**, The top 30 frequently mutated genes in *NF2*-SWN patients with paired blood samples.

Familial *NF2-*SWN is relatively rare, and we aim to comprehensively understand the somatic mutation landscape of familial *NF2-*SWN in order to identify potential therapeutic targets. We collected tumor samples from 7 cases of familial *NF2-*SWN, among which 3 cases had matched blood samples. Through WES of the *NF2-*SWN tumors from the 3 cases with blood samples as controls, we identified a total of 2648 genes that were mutated in at least one sample (Supplementary Table S1). Figure 3 presents a landscape of the somatic mutations in the samples with blood controls. The results indicated that missense mutations represented the predominant mutation type, with SNPs comprising over 80% of all mutations. Among these, the C>T substitution was identified as the most frequent SNV type, accounting for more than 75% (Figure 3A). Figure 3B illustrated the top 10 genes with the highest mutation counts, among which FLG and TTN exhibit the most frequent mutations, with 8 and 7 mutations respectively (Supplementary Table S1). Figure 3C displayed the top 30 genes with the highest mutation frequencies, 24 of which—including ATRX—were found to be mutated in all samples.

**Figure 4:**
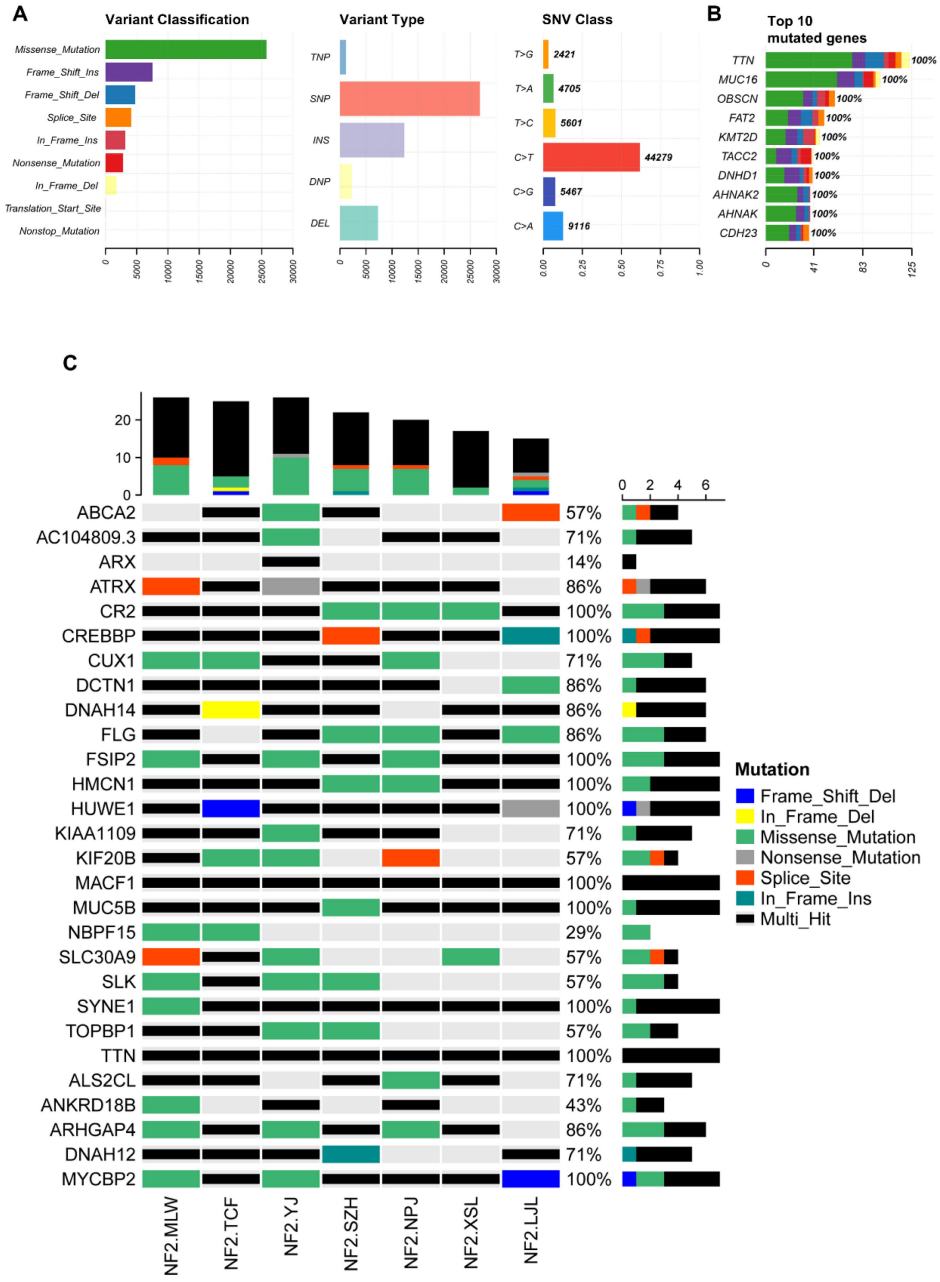
** The somatic mutation landscape of all seven *NF2*-SWN patients. A**, Landscape and counts of mutation types. **B**, The top 10 genes with the highest mutation counts. **C**, Mutation profiles of 30 previously identified genes in the background of the 1000 Genomes Project.

Due to limitations in sample collection, we were unable to obtain paired blood samples for all NF2-SWN tumors. Therefore, we analyzed seven *NF2*-SWN samples using data from the 1000 Genomes Project as a control^19^. As illustrated in Figure 4A, within the framework of the 1000 Genomes Project, gene mutations in familial *NF2-*SWN remained predominantly missense mutations and were primarily composed of SNPs, with C>T being the most common type of SNVs (>50%). This finding aligned with the results presented in Figure 3. Figure 4B displays the top 10 genes with the highest mutation counts in this background, among which TTN and MUC16 have the highest number of mutations. A total of 273 genes were mutated across all 7 samples (Supplementary Table S2). Considering that using paired blood samples as controls is more accurate, we validated the top 30 genes with the highest mutation frequency identified previously in the background of the 1000 Genomes Project. Most of these genes, such as ATRX, CR2, FLG, and FSIP2, still exhibited high mutation frequencies (Figure 4C), suggesting that mutations in these genes may play a role in the development and progression of familial *NF2-*SWN.

## Discussion

*NF2*-SWN presents a significant challenge in neurosurgery due to its low prevalence and poor prognosis. Currently, surgical resection is the primary treatment modality; however, the management of symptoms and postoperative complications necessitates the development of novel therapeutic approaches^20^-^22^. Despite early detection and surgical intervention, the prognosis for *NF2*-SWN is often poor, characterized by recurrent neurological tumors and hearing loss^21^, ^23^. Whole-exome sequencing demonstrates high sensitivity in identifying common, rare and low-frequency mutations, and effectively detecting most disease-associated mutations within exonic regions while requiring sequencing of only approximately 1% of the genome^24^, ^25^. Whole-exome sequencing is employed in the study of *NF2*-SWN to identify mutations within the population, thereby offering novel insights into the clinical treatment and prognosis of *NF2*-SWN. This research seeks to identify specific mutated genes in patients with a familial genetic history of *NF2*-SWN by utilizing WES, with the goal of elucidating the genetic or developmental mechanisms underlying the disease. Such findings have the potential to inform and innovate clinical treatment strategies for *NF2*-SWN.

Previous research has investigated the pathogenesis of *NF2*-SWN with a focus on gene expression. It is now widely recognized that the pathogenesis is attributed to the dysfunction of Merlin, a protein encoded by the tumor suppressor gene *NF2*. Merlin is part of the Ezrin/Radixin/Moesin (ERM) family of membrane cytoskeletal junction proteins. Furthermore, studies on skin tumors, vestibular schwannomas, and meningiomas in *NF2*-SWN patients have suggested that loss of heterozygosity due to *NF2* allele deletion and the inactivation of *NF2* gene transcription through hypermethylation may represent additional mechanisms of tumorigenesis^21^. However, these mechanisms have not been fully elucidated, indicating that the genetic study of *NF2*-SWN remains incomplete. In this study, genomic sequencing was performed on blood and tumor tissue samples, accompanied by an analysis of familial genetic history, leading to the identification of several genes of notable research interest. In this study, Whole-exome sequencing was conducted on tumor tissue samples using matched blood samples and the 1000 Genomes Project as control datasets. Integrated with family genetic history analysis, several genes of notable research significance were identified. These include TTN, which ranked among the top mutated genes in both analytical contexts; CR2, CREBBP, and FSIP2, which exhibited a 100% mutation frequency across both controls; and genes such as TTN, FLG, and HUWE1, which displayed a relatively high proportion of multi-hit mutations. These mutated genes may hold significant research implications for understanding the pathogenesis and progression of the disease.

The discovery of highly mutated genes such as TTN and FLG in this study warrants preliminary biological speculation. Currently, there are no studies have specifically investigated the involvement of TTN or FLG in *NF2*-SWN. TTN encodes a giant structural protein essential for sarcomere assembly in muscle cells and has been well-established as a key player in cardiomyopathy and heart failure^26^, ^27^. While its direct role in neuro-oncogenesis remains incompletely understood, a recent genomic analysis of craniospinal axis malignant peripheral nerve sheath tumors reported TTN mutations in up to 61% of cases^28^. This independent observation in nervous system tumors suggests that TTN mutations may potentially affect the biological behavior of schwannomas through mechanisms such as influencing cellular mechanical stability, signaling, or as yet unknown functions in Schwann cells. FLG (filaggrin) is mainly associated with skin barrier function and plays a significant role especially in skin diseases such as atopic dermatitis^29^. Its association with neuro-oncology is less direct. However, its recurrent appearance in our analysis prompts us to consider potential off-target effects or alterations in cell differentiation pathways that may interact with merlin loss of function. Future functional studies are crucial for validating these preliminary observations and elucidating any potential mechanistic links.

This study has several limitations that should be acknowledged. The most significant is the small sample size, particularly the limited number of familial cases with matched blood-tumor pairs (n=3), which is inherent to research on rare diseases like *NF2*-SWN. For the remaining four patients, we utilized the 1000 Genomes Project database as a control for somatic mutation analysis. Although this is a common strategy in genetic studies, it is theoretically less accurate than using patient-matched samples and may affect the precision of mutation screening. Consequently, the mutation frequencies reported here (e.g., in TTN, FLG) must be interpreted with caution. These findings are preliminary and hypothesis-generating, highlighting candidate genes that may contribute to *NF2*-SWN pathogenesis but require validation in a larger, independent cohort through future multi-center collaborations.

In conclusion, the specific genes discovered in this study provide potential avenues for further research. Genes such as TTN, CR2, FSIP2 and FLG may play a crucial role in specific aspects of disease progression, and it is worth exploring their specific functions and mechanisms in more detail.

## Supplementary Material

Supplementary tables.

## Figures and Tables

**Table 1:** Diagnosis criteria for *NF2*-related schwannomatosis, formerly known as NF2.

Diagnostic criteria for *NF2*-related schwannomatosis				
A diagnosis of *NF2*-related schwannomatosis (previously termed neurofibromatosis 2, NF2) can be made when an individual has one of the following:				
1. Bilateral vestibular schwannomas (VS)				
2. An identical *NF2* pathogenic variant in at least 2 anatomically distinct *NF2*-related tumors (schwannoma, meningioma, and/or ependymoma). (Note: if the variant allele fraction (VAF) in unaffected tissues such as blood is clearly <50%, the diagnosis is mosaic NF2-related schwannomatosis)				
3. Either 2 major or 1 major and 2 minor criteria as described in the following:				
Major criteria: • Unilateral VS • First-degree relative other than sibling with *NF2*-related schwannomatosis • 2 or more meningiomas (Note: single meningioma qualifies as minor criteria) • *NF2* pathogenic variant in an unaffected tissue such as blood (Note: if the VAF is clearly if the VAF is clearly <50%, the diagnosis is mosaic NF2-related schwannomatosis)				
Minor criteria: Can count >1 of a type (e.g., 2 distinct schwannomas would count as 2 minor criteria) • Ependymoma, meningioma (Note: multiple meningiomas qualify as a major criteria), schwannoma (Note: if the major criterion is unilateral VS, at least 1 schwannoma must be dermal in location) Can count only once (e.g., bilateral cortical cataracts count as a single minor criterion) • Juvenile subcapsular or cortical cataract, retinal hamartoma, epiretinal membrane in a person aged <40 years, meningioma Pattern of genetic changes in unaffected and tumor tissue in *NF2*-related schwannomatosis				
Gene locus	Unaffected Tissue	Tumor 1	Tumor 2	Comment
*NF2*				
Allele 1	PV1	PV1	PV1	Shared *NF2* pathogenic variant
Allele 2	WT	LOH or *NF2* PV2	LOH or *NF2* PV3	Tumor-specific partial loss of 22q in trans position or a different *NF2* somatic second PV in every anatomically unrelated tumor

**Table 2:** Clinical characteristics of all patients in this study

Characteristics	Number (cases)	Proportion (%)
**Total**	29	100
**Gender**		
Male	15	51.7
Female	14	48.3
**Age of onset**		
<40	27	93.1
≥ 40	2	6.9
**Initial symptoms**		
Tinnitus, hearing loss	21	72.4
Dizziness, headache	7	24.1
Limb weakness, walking instability	9	31
Loss of consciousness	1	3.4
**Family history**		
Familial	7	24.1
Sporadic	22	75.9
**Tumor number**		
Single	2	6.9
Multiple	27	93.1
**Pathology**		
Schwannoma	23	79.3
Meningioma	8	27.6
Perineurioma	1	3.4
**Tumor location**		
Supratentorial	18	60
Subtentorial	29	96.7
Intraspinal	5	16.7

## Abbreviations

NF2: Neurofibromatosis type 2
*NF2-*SWN: *NF2*-related schwannomatosis
SNV: Single nucleotide variation
SNPs: Single nucleotide polymorphisms
INDELs: Insertions/deletions
WES: Whole-exome sequencing
ERM: Ezrin/Radixin/Moesin
